# The Role of Epoxyeicosatrienoic Acids in Cardiac Remodeling

**DOI:** 10.3389/fphys.2021.642470

**Published:** 2021-02-24

**Authors:** Jinsheng Lai, Chen Chen

**Affiliations:** Division of Cardiology, Tongji Hospital, Tongji Medical College and Hubei Key Laboratory of Genetics and Molecular Mechanisms of Cardiologic Disorders, Huazhong University of Science and Technology, Wuhan, China

**Keywords:** EET, AA, cytochrome P450 epoxygenases, epoxyeicosatrienoic acids, cardiac remodeling, cardiovascular disease, heart failure, drug development

## Abstract

Epoxyeicosatrienoic acids (EETs) are metabolites of arachidonic acid by cytochrome P450 (CYP) epoxygenases, which include four regioisomers: 5,6-EET, 8,9-EET, 11,12-EET, and 14,15-EET. Each of them possesses beneficial effects against inflammation, fibrosis, and apoptosis, which could combat cardiovascular diseases. Numerous studies have demonstrated that elevation of EETs by overexpression of CYP2J2, inhibition of sEH, or treatment with EET analogs showed protective effects in various cardiovascular diseases, including hypertension, myocardial infarction, and heart failure. As is known to all, cardiac remodeling is the major pathogenesis of cardiovascular diseases. This review will begin with the introduction of EETs and their protective effects in cardiovascular diseases. In the following, the roles of EETs in cardiac remodeling, with a particular emphasis on myocardial hypertrophy, apoptosis, fibrosis, inflammation, and angiogenesis, will be summarized. Finally, it is suggested that upregulation of EETs is a potential therapeutic strategy for cardiovascular diseases. The EET-related drug development against cardiac remodeling is also discussed, including the overexpression of CYP2J2, inhibition of sEH, and the analogs of EET.

## Introduction

Cardiovascular disease (CVD) is recognized as a leading cause of mortality worldwide (Roth et al., 2015). Heart failure (HF) is the final stage of CVD, which has become a burgeoning problem that tortures about 23 million people (Mozaffarian et al., 2015; Murphy et al., 2020). Exploring the pathophysiological etiologies of HF is essential to identify novel therapeutic strategies. Dramatically, during the progress of HF, cardiac remodeling is one of the key pathophysiological processes. It is a maladaptive change including changes in wall thickness, ventricular volumes, and cardiac mass to various internal and (or) external pathological stimulus, which ultimately causes noncompensatory HF (Opie et al., 2006; Braunwald, 2013; Yang et al., 2020).

Up to now, the underlying mechanisms of cardiac remodeling are not completely elucidated; several pathological changes are involved, including cardiac hypertrophy, cardiomyocytes apoptosis, cardiac fibrosis, inflammation, oxidative stress, and endothelial dysfunction (Wu et al., 2017; Zhou et al., 2019). Traditionally, it is believed that cardiac remodeling is irreversible. Once it develops into HF, there is no effective therapy. Thus, cardiac remodeling is becoming an important therapeutic target for HF (Kim et al., 2018). The overall goal of HF therapy is to ameliorate symptoms, decrease hospitalization rates, and prevent premature death. Although there are several drugs targeting HF, such as angiotensin-converting enzyme (ACE) inhibitors, beta blockers, and mineralocorticoid receptor antagonists, which are able to slow down the progress of cardiac remodeling, the morbidity remains at a very high level (Tham et al., 2015). Thus, more therapeutic targets are urgently needed.

Recently, epoxyeicosatrienoic acids (EETs) have been reported as novel agents against cardiac remodeling (Alsaad et al., 2013; Althurwi et al., 2015; Romashko et al., 2016). EETs are the metabolites of arachidonic acids (AAs) through the cytochrome P450 (CYP) epoxygenase pathway (Capdevila et al., 2000), and it has been proved that the levels of EETs could be elevated by several strategies. *In vivo* and *in vitro* studies suggested that EETs acted as protective effectors against cardiac remodeling and ameliorated HF (Xu et al., 2011). Meanwhile, the levels of EETs could be elevated by several approaches, for example, soluble epoxide hydrolase (sEH) inhibitors. Thus, it could be a promising therapy target. Of course, more work needs to be done to translate it from experimental studies to the clinic. In the present review, we highlight the effects of EETs on cardiac remodeling in CVD.

## EETs and Its Effects in CVD

**FIGURE 1:**
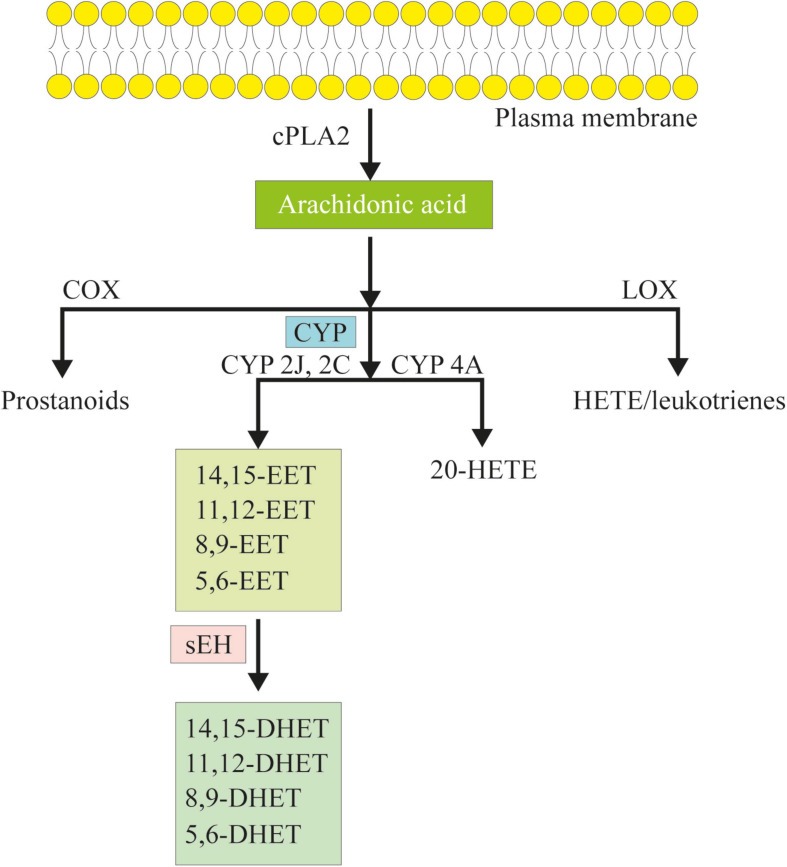
The cascade of arachidonic acid (AA). AA is a polyunsaturated omega-6 fatty acid which is released from the membrane phospholipids in the presence of phospholipase a2 (cPLA2). AA can be metabolized to eicosanoids through three major pathways: the cyclooxygenase (COX) pathway, the lipoxygenase (LOX) pathway, and the cytochrome P450 (CYP) pathway. In the CYP pathway, AA is converted to epoxyeicosatrienoic acids (EETs) and 20-hydroxy eicosatetraenoic acid (HETE) by CYP epoxygenases and CYP ω-hydroxylases. Following, EETs can be hydrated *in vivo* to dihydroxyeicosatrienoic acids (DHETs) by soluble epoxide hydrolase (sEH).

CYP is a superfamily of membrane-bound, NADPH-dependent monooxygenases, which plays a vital role in the oxidation of both xenobiotics and endogenous compounds (Aliwarga et al., 2018). Up to now, 57 different genes, arranged in 18 families and 43 subfamilies, were found in the human CYP superfamily (Guengerich, 2006). Among them, the CYP2J and CYP2C families are the most important enzymes in the synthesis of EETs. CYP2J2 is the only human CYP2J epoxygenase, which is dominantly expressed in the heart, especially in the endothelium, and is receiving increasing attention due to its metabolites (Wu et al., 1996). CYP2J2 converted AA into four regioisomeric EETs, including 5, 6-, 8, 9-, 11, 12-, and 14,15-EET (Zeldin, 2001; Rand et al., 2017). Among them, more than 90% of the products are 14,15-EET and 11,12-EET (Sudhahar et al., 2010). Once synthesized, EETs can be hydrated *in vivo* into dihydroxyeicosatrienoic acids (DHETs) by sEH, especially 11,12-EET and 14,15-EET (Figure 1; Zeldin et al., 1995; Zeldin, 2001; Sudhahar et al., 2010). sEH is the production of gene EPHX2, which is ubiquitous in human and animal tissues. So far, sEH has been identified as an important target to upregulate the level of EETs *in vivo* (Yu et al., 2000). In addition, AA could be metabolized into prostanoids by cyclooxygenase (COX) or hydroxy eicosatetraenoic acid (HETE) and leukotrienes by lipoxygenase (LOX) (Figure 1; Zeldin, 2001).

In 1981, Capdevila et al. (1981) firstly reported that AA could be metabolized to EETs by CYP in the liver. Since then, plenty of studies have demonstrated that EETs played important roles in several diseases, such as kidney diseases (Hye Khan et al., 2016; Deng et al., 2017; Wang et al., 2019), neurodegenerative diseases (Atone et al., 2020; Pallas et al., 2020), rheumatic arthritis (Hoxha, 2018; Hoxha and Zappacosta, 2020), and especially CVD (Xu et al., 2011; Chen and Wang, 2013).

In 1980s, EETs were reported to have vasodilator effects and could lower blood pressure in animals (Zeldin, 2001). Since then, accumulated data revealed that EETs showed protective effects against various CVDs. It has been well established that EETs are potent vasodilators *in vivo*, which are independent of nitric oxide (NO) in response to bradykinin (Imig et al., 2001). Furthermore, evidence from various studies showed that EETs acted as endothelium-derived hyperpolarizing factor (EDHF), which mediated the vasodilation of vascular smooth muscle by activating Ca^2+^-activated K^+^ channels (Campbell and Fleming, 2010). In the animal models of hypertension, synthesis of EETs was inhibited (Capdevila et al., 2007), and EET levels were upregulated by sEH inhibitors or overexpression of CYP genes, which in turn decreased blood pressure *in vivo* (Imig et al., 2002; Lee et al., 2010). Data from our laboratory also confirmed these effects of 14,15-EET in spontaneously hypertensive rats by enhancing atrial natriuretic peptide (ANP) (Xiao et al., 2010). Besides, EET analogs that mimic the endogenous EETs showed blood pressure-lowering effects in spontaneously hypertensive rats and angiotensin-associated hypertension (Imig et al., 2010; Hye Khan et al., 2014). These beneficial roles of EETs are attributed to anti-inflammation, vasodilation, and natriuresis (Imig, 2019).

Atherosclerosis is defined as a chronic vascular inflammatory disease induced by chronic inflammation and abnormalities in cholesterol metabolism. It is the main pathologic change in coronary artery disease (CAD) and myocardial infarction (MI), which are responsible for majority of cardiac death. Interestingly, EETs attenuated the expression of pro-inflammatory genes and proteins (Schmelzer et al., 2005), while sEH inhibition ameliorated lipid metabolism disorder by reducing cholesterol and low-density lipoprotein (LDL) levels and increasing high-density lipoprotein (HDL) levels (Zhang et al., 2009; Wang et al., 2010). Thus, EETs could act as anti-atherosclerosis factors in CAD and MI. Substantial studies have proved that EETs prevented atherosclerosis in several models, including ApoE^–/–^ mice and high-fat diet (HFD)-induced models (Wang et al., 2010; Liu et al., 2016). Besides, Oni-Orisan et al. (2016) reported that lower EET levels including 8,9-EET, 11,12-EET, and 14,15-EET were observed in obstructed CAD patients compared with patients with no apparent CAD, which was consistent with the results reported by Theken et al. (2012).

Moreover, EETs offer protective effects on MI. Previous studies provided evidence that treatments with sEH inhibitors could reduce the infarct size and chronic cardiac remodeling post MI, which was beneficial in preventing electrical remodeling and cardiac arrhythmias post MI, as well as reducing inflammation (Li et al., 2009). Concomitantly, EET agonist, e(*S*)-2-(11-(nonyloxy)undec-8(*Z*)-enamido)succinic acid (NUDSA), restored cardiac function, promoted angiogenesis, and ameliorated fibrosis in MI mice induced by left anterior descending ligation (Pullen et al., 2020). These effects were mediated by increasing the canonical Wnt1 signaling cascade with the subsequent increase in heme oxygenase-1 (HO-1) (Cao et al., 2015).

HF is the end-stage of all kinds of CVD due to cardiac remodeling. As mentioned above, once HF progressed, it is always irreversible and lacks effective treatments. Researches showed that elevation of EETs might represent a promising therapeutic strategy combating HF. In a recent review including various studies employing HF animal models, consistent results were observed, wherein upregulation of EETs by sEH inhibitors improved cardiac function in different HF models (Qiu et al., 2011). In line with that, our laboratory also proved that CYP2J2-derived EETs attenuated cardiac function detected by echocardiography and invasive pressure–volume analysis (Wang et al., 2014). It is believed that inhibition of endoplasmic reticulum (ER) stress and oxidative stress was involved in the effects of overexpression of CYP2J2 and exogenous 11,12-EET, which finally ameliorated cardiac hypertrophy (Wang et al., 2014).

Nevertheless, it is evident that inflammation is present in HF (Shirazi et al., 2017). In HF models induced by isoprenaline (ISO) (Harrison and Cai, 2003) or angiotensin II (Ang II), the expression levels of inflammatory cytokines were significantly increased as a result of NF-κB pathway activation, while overexpression of CYP2J2 reduced the levels of inflammatory cytokines and improved cardiac function (Yang et al., 2015).

Collectively, EETs are metabolites of AA via the CYP pathway and play a vital role in CVD, such as hypertension, CAD, and HF. However, more studies are needed to elucidate the underlying mechanisms and further translation to clinical use.

## The Role of EETs in Different Models of Cardiac Remodeling

Cardiac remodeling is an adaptive process to the cardiac overload caused by different stimuli. Typically, it could be divided into two types: one is pressure overload, while another is volume overload. In pressure overload diseases, such as hypertension, the cardiomyocytes increase in thickness more than in length to reduce the ventricular wall stress. Thus, the heart develops concentric hypertrophy with wall thickness. In volume overload diseases, such as MI or dilated cardiomyopathy, cardiomyocytes might reduce and rearrange with ventricular dilation. Therefore, the heart develops eccentric hypertrophy and ventricular dilation with lengthening of cardiomyocytes as a result of decreased cardiomyocyte number and rearrangement of surviving cardiomyocytes.

Actually, there might not be only two patterns of remodeling existing. For example, in HF induced by hypertension, at the early stage, there is mainly pressure overload, which makes the heart undergo hypertrophy, while in the late stage, the heart develops eccentric hypertrophy, so volume overload will also happen. These two patterns of cardiac remodeling are related closely. Nonetheless, there are differences between the two models, including different pathological changes and mechanisms (Toischer et al., 2010; You et al., 2018). In pressure overload heart, fibrosis and apoptosis are more significant with activation in calcium/calmodulin-dependent protein kinase II-dependent altered calcium cycling, whereas angiogenesis was more frequently in the volume overload model with the activated AKT pathway (Toischer et al., 2010; Kim et al., 2019).

Numerous studies have reported that EETs ameliorated cardiac remodeling, including both pressure overload- and volume overload-induced cardiac remodeling. In the following section, we will review the roles of EETs in different models of cardiac remodeling.

### EETs in Pressure Overload-Induced Cardiac Remodeling

Pressure overload is the main inducible factor of cardiac remodeling. It could be involved in several diseases, such as hypertension, aortic stenosis, and aortic coarctation, which finally results in HF. There are several models employed, and EETs showed a protective effect on pressure overload-induced cardiac remodeling.

In the pressure overload-induced cardiac remodeling, drugs, such as Ang II, ISO, and endothelin 1 (ET-1), were always used. Among them, Ang II is the most common one, which acts as a potent vessel constrictor that elevates blood pressure (afterload to the heart). Interestingly, it is demonstrated that the expression of sEH was upregulated, and the level of EETs was downregulated in Ang II-induced hypertension (Ai et al., 2007; Pang et al., 2011). Given that, what would happen if the expression of EETs was restored? sEH is well known as a hydrolase of EETs. In Ang II-treated hearts, the size of cardiomyocytes and the expression of hypertrophy markers, including the atrial natriuretic factor and myosin heavy chain, were significantly elevated, while administration with a potent sEH inhibitor, AEPU, prevented this pathogenesis (Ai et al., 2009). In addition, CYP2J2-derived EETs also played a protective role in Ang II-induced cardiac remodeling. In mice treated with Ang II, cardiac hypertrophy was verified by echocardiography and invasive pressure–volume analysis, with increases in the ratio of heart weight to body weight and cardiomyocyte apoptosis, as well as ER stress. Overexpression of CYP2J2 showed an elevation in 14,15-DHET, the metabolite of 14,15-EET, which inhibited the expression of ER stress molecules, GRP-78, CHOP, and cleaved ATF-6. As a result, CYP2J2 transgenic mice showed a reversed heart function in the presentation of Ang II (Wang et al., 2014). Our recent data also showed that 11,12-EET and 14,15-EET ameliorated cardiac remodeling by attenuating oxidative stress via PPAR-γ activation (He et al., 2015) and by suppressing transmission of pro-inflammation from cardiomyocytes to macrophages in the heart (Yang et al., 2015).

The transverse aortic constriction (TAC) model is another model commonly used to investigate cardiac remodeling upon pressure overload. In 1991, Rockman et al. (1991) first reported this model to study the mechanism underlying cardiac hypertrophy. At the early stage, the heart develops cardiac hypertrophy in the presence of dramatically increased afterload, while it finally causes HF with eccentric hypertrophy (Zi et al., 2019; Bosch et al., 2020). Cardiac remodeling is involved throughout the process, and the calcineurin-NFAT pathway is one of the most important signaling pathways (Rohini et al., 2010). A recent study carried out by Li et al. proved it again in mice. Mice with hypertrophy induced by TAC appeared with an impaired cardiac function and abundant fibrosis subsequent to the activation of the calcineurin/NFAT and TGF-β/Smad pathway. However, these effects could be suppressed by overexpression of CYP2J2 mediated by the recombinant adeno-associated virus (rAAV) and treatment with 11,12-EET (Li et al., 2020a). Coincidentally, TAC induced a severe hypertrophic response and decline of cardiac function in wild-type mice, while these features were ameliorated in CYP2J2 transgenic mice. Dramatically, mice with an overexpression of CYP2J2 showed a significant decrease in mortality after TAC (6%) compared with the wild-type mice (42%) (Westphal et al., 2013).

Furthermore, the effects of EETs by inhibition or deletion of sEH are explored in the TAC-induced cardiac remodeling model. The human sEH is the product of gene EPHX2 with 19 exons on chromosome 8 (Sandberg and Meijer, 1996; Qiu et al., 2011). Four weeks after TAC, the EPHX2^–/–^ mice did not present a hypertrophic phenotype compared with the control mice, indicated by having no change in the ratio of heart weight to body weight and in wall thickness as evaluated by echocardiography (Zhang et al., 2014). This effect was related to the deletion of the sEH enzyme, while inhibition of sEH remained controversial. Xu et al. (2006) reported that treatment with sEH inhibitors, AEPU or AUDA, prevented the development of cardiac hypertrophy after 3 weeks of TAC in mice. Furthermore, in the presence of cardiac hypertrophy by TAC, treatment with AEPU or AUDA for another 3 weeks showed a reversible effect against cardiac hypertrophy. It was demonstrated that these compounds potently block the NF-κB pathway activation in cardiomyocytes (Xu et al., 2006). However, another study employed two novel synthesis compounds, GSK2188931 and GSK2256294, which showed a potent inhibition on sEH by increasing the leukotoxin (LTX)/LTX diol ratio and reducing plasma 14,15-DHET. Unexpectedly, GSK2188931 and GSK2256294 exhibited no influence on mortality, tissue weights, hypertrophic biomarkers, fibrosis level, and cardiac function and morphology after TAC (Morgan et al., 2013). These disparate results indicate that the role of EETs and sEH in cardiac remodeling is complex and model dependent, and the physical and chemical properties of different chemical structures of sEH inhibitors are also different, which needs further work to explore the underlying mechanism.

### EETs in Volume Overload-Induced Cardiac Remodeling

Other than pressure overload-induced cardiac remodeling, volume overload-induced cardiac remodeling is characterized by wall thinning and chamber dilatation with cardiomyocyte lengthening. It is involved in several diseases, such as aortic regurgitation (AR) and mitral regurgitation (MR). In rodents, aortocaval fistula (ACF) is most widely used for inducing volume overload. It is operated by puncturing the shared midwall between the aorta and inferior vena cava with a sharp needle (Liu et al., 1991). There are three stages of HF progression in response to volume overload: acute stress (12 h–7 days), compensatory remodeling (3–10 weeks), and decompensated HF (>15 weeks) (Hutchinson et al., 2010).

The effects of EETs in ACF-induced volume overload-related cardiac remodeling have been studied previously (Cervenka et al., 2015a, b; Sporkova et al., 2017; Kala et al., 2018; Vackova et al., 2019a, b). It is demonstrated that the level of EETs is downregulated in the heart of ACF animals, while the expression of sEH is upregulated (Cervenka et al., 2015a, b). Restoration of EETs by sEH inhibition showed a protective effect against chronic HF by 10 weeks in Ren-2 transgenic rats combined with ACF. The conclusion was confirmed by pressure–volume analyses. It is shown that sEH inhibitor treatment significantly lowered left ventricular peak pressure in chronic HF (CHF) animals, which finally remarkably increased survival rate from 14 to 41% (Cervenka et al., 2015a). However, in Han SD rats, treatment with sEH inhibitors did not prevent the development of CHF, and these rats presented with a similar death curve to that of control groups (Cervenka et al., 2015b). The only difference between the studies is the rat strain. One is a Ren-2 transgenic rat and another is a Han SD rat. The Ren-2 transgenic rat is a model of Ang II-related hypertension, which means that the different effects of sEH inhibition between the Ren-2 transgenic and Han SD rats revealed the importance of the interaction of hypertension, renin–angiotensin system (RAS), and CYP-derived metabolites in the progression of CHF-related mortality. Dramatically, a combination of ACE inhibitor and sEH inhibitor did not elicit a significant elevation in survival rate (53%), while ACE inhibitor alone showed a significant effect on the course of CHF with a survival rate of 84% (Kala et al., 2018). Since these results lack probable explanations, further studies are needed to address these issues.

It is known that MI contributes to the development of cardiac remodeling and HF. MI-related HF is viewed as a kind of volume overload cardiac remodeling because it is characterized with ventricular dilation and decline in ejection fraction (Pfeffer and Braunwald, 1990). Once MI occurred, cardiomyocytes died due to inadequate blood supply. Although ischemia induces angiogenesis in the infarcted area, this process is insufficient (van der Laan et al., 2009). Thus, drugs promoting angiogenesis are thought to be effective in MI. Dozens of studies have proved that EETs are potent inducible factors of angiogenesis (Pozzi et al., 2005; Fleming, 2007; Rand et al., 2019). Theoretically, elevation of EETs can be an important strategy of MI-induced cardiac remodeling. Thus, scientists did make great efforts in exploring the effects of EETs on MI-related cardiac remodeling.

Recently, evidence revealed that overexpression of CYP2J2 and 11,12-EET significantly increased vascular endothelial growth factor (VEGF), which enhanced myocardial angiogenesis and improved cardiac function in MI-induced HF (Zhao et al., 2018). Using the same transgenic mice, Aliwarga et al. (2020) reported that improved cardiac performance was observed in the CYP2J2 transgenic mice following MI. Besides, transgenic mice ameliorated myocardial remodeling induced by MI, evidenced by higher fractional shortening, smaller infarct size, lower reactive oxygen species (ROS) formation, reduced fibrosis and apoptosis, and lower pulmonary edema. Interestingly, treatment with EET agonist (Cao et al., 2015) or EET analog (Neckar et al., 2019) both increased the expression of HO-1 and attenuated post-MI cardiac remodeling. HO-1 is a cytoprotective enzyme, which protects the heart from remodeling after MI (Lakkisto et al., 2011).

More convincing studies were conducted with sEH inhibitors, and similar results were observed (Seubert et al., 2006; Zhao et al., 2012). Administration with TPPU, a potent sEH inhibitor-activated AKT pathway, promoted angiogenesis and restored blood supply, which finally inhibited ventricular enlargement and improved cardiac function after MI (Guo et al., 2018). Oxidative stress is involved in cardiac remodeling and responsible for apoptosis in the stressed heart. In an established chronic HF model by coronary ligation, the heart function was reduced, as evidenced by impaired parameters in LV hemodynamics, increased oxidative stress with increasing ROS level, and reduced GSH-to-GSSG ratio. Fortunately, these impairments induced by MI could be reversed by sEH inhibition with AUDA (Merabet et al., 2012). EPHX2 has been recognized as an HF susceptibility gene. In ischemic HF patients, the expression of EPHX2 was significantly decreased compared with that in the control group who maintained a high level of 14,15-EET (Monti et al., 2008). However, sEH deletion activated cardiac ATP-sensitive K^+^ channels, enhanced L-type calcium currents, and improved cardiac function after ischemia/reperfusion injury (Seubert et al., 2006). Consistently, sEH deletion in EPHX2-null mice limited cardiac functional decline following MI in both aged and young mice following MI by preserving mitochondrial bioenergetics (Jamieson et al., 2017b). These effects were in proportion to the inhibition tensity of sEH. In EPHX2-null mice, plasma levels of 8, 9-, 11, 12-, and 14,15-DHET were reduced by 38, 44, and 67%, while in dual EPHX1- and EPHX2-null mice, the levels of the DHETs were reduced by 100, 99, and 96%, respectively. In addition, compared to EPHX2-null mice, dual EPHX1- and EPHX2-null mice showed a better heart function recovery induced by MI (Edin et al., 2018).

Collectively, EETs played important roles in both pressure overload- and volume overload-induced cardiac remodeling, and the underlying mechanisms seems to be different from each other (Table 1). Although EETs seem to have similar effects against both types of cardiac remodeling, few studies focused on the different effects. The effects of EETs on cardiac remodeling could be affected by several factors including gender. It is well known that gender is an important factor in CVD. The differences between male and female in cardiovascular health could be affected by many factors, such as sex hormones, gene expression, and sociocultural aspects. It has been reported that the level of EETs, especially 11,12-EET in female mice, was significantly higher than that in male mice when challenged by MI, which was beneficial to the female mice in cardiac recovery (Pullen et al., 2020). Interestingly, sEH gene expression was also different between genders. Qin et al. (2016) reported that the expression of sEH in female mice was significantly lower compared to that in males. These might also contribute to the different responses between males and females during cardiac diseases. It indicates that deeper studies need to be carried out to understand the underlying mechanism.

**TABLE 1:** The mechanisms involved in the protective effects of EETs in cardiac remodeling.

**Pressure-overload related cardiac remodeling**
Activation of PPARγ pathway
Inhibition of ER stress
Prohibiting the transmission of pro-inflammation
Inhibition of calcineurin/NFAT pathway
Reducing fibrosis by inhibiting TGF-β/Smad pathway
**Volume-overload related cardiac remodeling**
Decreasing apoptosis
Induction of VEGF
Activation of AKT pathway
Increasing the expression of HO-1
Regulating the expression of ion channels
Inhibition of oxidative stress

## The Role of EETs in the Pathological Changes of Cardiac Remodeling

Cardiac remodeling mainly refers to a maladaptive process to pressure overload or volume overload, which is characterized by myocardial hypertrophy, inflammation, apoptosis, fibrosis, and vascular dysfunction. During the processes, these pathogeneses interact with each other and finally cause HF (Zhou et al., 2019). EETs have been reported to have protective factors against cardiac remodeling, including these pathogeneses.

### EETs and Myocardial Hypertrophy in Cardiac Remodeling

Myocardial hypertrophy is the major pathologic change in cardiac remodeling. The role of EETs in myocardial hypertrophy has been reviewed previously (Wang et al., 2013). Xu et al. reported that increasing the level of EETs by sEH inhibitors could prevent pressure-induced cardiac hypertrophy. In addition, inhibition of sEH reversed the development of cardiac hypertrophy caused by chronic pressure overload (Xu et al., 2006). In an *in vitro* study, ISO induced hypertrophic phenotype in cardiomyocytes by elevating the hypertrophic markers (ANP and BNP), which could be inhibited by sEH inhibition (Althurwi et al., 2013, 2015).

Moreover, CYP2J2-derived EETs also showed protective effects on cardiac hypertrophy (Alsaad et al., 2013). In the ISO-induced cardiac hypertrophy model, hypertrophy was initiated 72 h after ISO treatment. In the hypertrophic heart, the levels of EETs were observed, while restoring EET levels by overexpression of CYP2J2 prevented the initiation of cardiac hypertrophy through NF-κB-mediated mechanism (Althurwi et al., 2015). However, Tse et al. (2013) reported that the expression of CYP enzymes was increased after treatment with ISO, which also mediated the increase in the cell surface area. Interestingly, 14,15-EET significantly attenuated the ISO-mediated induction of cardiac hypertrophy. Coincidentally, our laboratory also showed that overexpression of CYP2J2 and 11,12-EET attenuated cardiac hypertrophy elicited with Ang II, which was mediated through the activation of AMPK-α2 (Wang et al., 2016).

### EETs and Apoptosis in Cardiac Remodeling

A large number of experimental data support the presence of apoptosis in the progress of HF. Apoptosis is also called programmed cell death, which can be found in all the cardiac remodeling models, such as aortic constriction model and coronary artery occlusion model (Chen and Tu, 2002). It has been demonstrated that the resulting reduction of cardiomyocytes would finally lead to HF (Olivetti et al., 1997; Williams, 1999). Increasing data revealed that EETs inhibited apoptosis mediated by different pathways in cardiac remodeling. Oxidative stress and ER stress are initiators of cardiomyocyte apoptosis. In HF models induced by Ang II, increased levels of oxidative stress and ER stress were noticed in the heart compared with the control animals, which was responsible for the increased apoptosis level confirmed by TUNEL staining. Excitingly, all these effects were abolished by overexpression of CYP2J2, while 14,15-EET significantly ameliorated ER stress and subsequently apoptosis in cultured cardiomyocytes (Wang et al., 2014). In another *in vitro* study, 11,12-EET is involved in cardioprotection effects by inhibiting apoptosis via a caspase-dependent pathway, indicated by the ratio change of apoptotic protein expression (Bcl2 and Bax) and activation of pro-apoptotic caspase-3 (Wang et al., 2012). Moreover, in ethanol-induced HF models, cardiac remodeling was evidenced by cardiac dilation and dysfunction. In addition, the levels of apoptosis and oxidative stress were increased in ethanol-treated hearts, while overexpression of CYP2J2 and exogenous 11,12-EET ameliorated apoptosis and oxidative stress in ethanol-induced HF (Zhou et al., 2018). The phosphatidylinositol 3-kinase (PI3K)/AKT pathway is one of the strongest anti-apoptotic signaling systems. In an *in vitro* study, cardiomyocytes were subjected with hypoxia/anoxia to mimic ischemia/reperfusion injury. Abundant apoptosis of cardiomyocytes was recorded via inhibition of the PI3K/AKT pathway, indicated by activation of caspase 9 and caspase 3. Treatment with EETs (8,9-EET, 11,12-EET, and 14,15-EET) significantly increased the phosphorylation level of AKT and reduced the cardiomyocyte’s apoptosis level (Dhanasekaran et al., 2008).

Despite the apoptosis of cardiomyocytes, the apoptosis of endothelial cells in the heart plays a vital role in cardiac remodeling. In adult mice, endothelial cells accounted for 64% of non-cardiomyocytes (Pinto et al., 2016). Endothelial cells participate in the regulation of cardiac remodeling after pathological stress via secreting various biological molecules, such as adhesion molecules (ICAM-1 and tenascin-C) and angiogenetic factors (VEGF and IGF) (Yang et al., 2020). However, endothelial cells exhibited apoptosis when exposed to stimulus. TNF-α induced apoptosis in endothelial cells was analyzed with flow cytometry after Annexin V/PI staining, accompanied with activation of caspase 3 and downregulation of Bcl-2. CYP2J2 overexpression significantly inhibited caspase 3 activity and downregulated Bcl-2 expression. The anti-apoptotic effects of CYP2J2 overexpression in endothelial cells were attenuated by activation of the PI3K/Akt and inhibition of MAPK signaling pathways (Yang et al., 2007).

Besides, the cross talk between the endothelium and cardiomyocytes has been studied in EETs against apoptosis. As reported, the cardiomyocytes presented with apoptosis after ischemia. Interestingly, CYP2J2-specific overexpression in endothelial cells showed protective effects from apoptosis of cardiomyocytes after ischemia, which finally ameliorated cardiac function (Zhao et al., 2018).

### EETs and Fibrosis in Cardiac Remodeling

Fibrosis always occurs in both cardiac remodeling models. In pressure overload-induced cardiac remodeling, fibrosis is mainly presented in interstitial substance without cardiomyocyte deletion. However, in volume overload-induced cardiac remodeling, the cardiomyocytes were dramatically reduced, and fibrosis is evoked, especially in MI.

Four weeks after TAC, the mice developed maladaptive cardiac hypertrophy with abundant fibrosis and increased collagen level in the heart. Overexpression of CYP2J2 significantly inhibited fibrosis in TAC mice and protected against HF (Li et al., 2020a). In fibrosis, the TGF-β/Smad pathway is one of the most studied pro-fibrosis signaling pathways. It was proven that the TGF-β/Smad pathway was involved in the process (Li et al., 2020a). They also found that similar effects were observed in the abdominal aortic constriction model, which is another pressure overload-induced cardiac remodeling model (Li et al., 2020b). Besides, Ang II-induced cardiac remodeling is always accompanied by cardiac fibrosis. He et al. exposed CYP2J2 transgenic mice to Ang II treatment for 2 weeks continuously to induce HF. Results show that Ang II elicited cardiac fibrosis by enhancing collagen accumulation and increasing expression of α-SMA and collagen I, which are the markers of cardiac fibrosis. Overexpression of CYP2J2 and 11,12-EET inhibited cardiac fibrosis via inhibition of Gα12/13/RhoA/ROCK signaling (He et al., 2017). At the same time, the effects against fibrosis in Ang II-induced HF were confirmed by Yang et al. (2015), which were mediated by suppressing the transmission of pro-inflammation from cardiomyocytes to macrophages in the heart.

Ischemic cardiomyopathy is a common cause of HF, and fibrosis is involved in ischemia-induced cardiac remodeling. The key roles played by EETs in MI-related cardiac remodeling have been reviewed previously. Treatment with sEH inhibitor limited the cardiac fibrosis after MI, which is dependent on the drug dose. The higher the dose used, the smaller the fibrosis area observed in the heart (Guo et al., 2018). Besides, both EET agonist (Cao et al., 2015) and EET analog (Neckar et al., 2019) showed anti-fibrosis effects determined by histochemical stain.

### EETs and Inflammation in Cardiac Remodeling

Inflammation is an important pathological process of diseases, including HF. It is evident that inflammation is present in both acute and chronic HF, and a higher level of pro-inflammation factors is related to poorer prognosis. Thus, exploring anti-inflammatory therapy is a new target of HF. It was found that EETs are important anti-inflammatory mediators (Inceoglu et al., 2007). Node et al. (1999) first reported the anti-inflammatory effects of EETs in response to several inflammatory mediators, such as TNF-α and interleukin 1α (IL-1α), by inhibition of the NF-κB pathway. NF-κB is a nuclear transcription factor that regulates expression of a plenty of genes that are critical for inflammation. Both 11,12-EET (Bystrom et al., 2011) and 14,15-EET (Morin et al., 2008) inhibited NF-κB activation *in vitro*. Our laboratory also revealed that in challenge to Ang II, mice exhibited cardiac remodeling with NF-κB pathway activation, while CYP2J2 transgenic mice ameliorated cardiac function with reduced NF-κB p65 nuclear translocation (He et al., 2015). It is well established that PPAR-γ is a key factor in the anti-inflammation process. EETs have been reported as ligands for PPAR-γ and increased PPAR-γ transcription activity in endothelial cells and 3T3-L1 preadipocytes (Liu et al., 2005). The anti-inflammation effects of EETs could be abolished by PPAR-γ antagonist both *in vivo* and *in vitro* (Liu et al., 2005; He et al., 2015). Besides, a PPAR-γ-independent pathway was also involved in Ang II-related inflammation. In our recent study, Ang II induced the activation of the JAK2/STAT3 pathway and subsequently resulted in inflammation and fibrosis of the aorta. Overexpression of CYP2J2 induced the expression of SOCS3, which inhibited the activation of the JAK2/STAT3 pathway independent of the PPAR-γ/NF-κB pathway (Zhou et al., 2016).

HO-1 is the rate-limiting enzyme in the catabolism of heme. It plays an important role in the amelioration of cardiac remodeling and inflammation. The overlapping effects of EETs and HO-1 led researchers to figure out the relationship between them. Several studies were carried out, and results showed that EETs could be an inducer of HO-1 expression (Sacerdoti et al., 2007; Li et al., 2009; Aliwarga et al., 2020). Moreover, in obesity-related cardiomyopathy, proinflammatory adiponectin and damaged cardiac function were observed, while treatment with EET analog ameliorated the expression of proinflammatory adiponectin and prevented HF. These effects may be due to the induction of HO-1 by the EET analog (Cao et al., 2017).

Other studies indicated that sEH was a target for the inhibition of inflammation (Schmelzer et al., 2005). AUDA, a potent sEH inhibitor, protected the mice from inflammation via inhibiting the NF-κB pathway (Liu et al., 2005). The effects were also observed in cardiac remodeling models. Stevenson et al. evaluated the inflammation cytokines (including MMP9, CCL5, CCL4, and IL-16) in human ischemic cardiomyopathy, results showed that these inflammation cytokines were upregulated in ischemic cardiomyopathy hearts in both mRNA and protein levels. In an *in vivo* study, HF induced by LAD ligation was elicited, and the inflammation cytokine CCL5 was markedly increased after LAD ligation in the hearts. Importantly, the expression of sEH in ligation hearts was significantly greater than that in hearts from wild-type mice. In addition, the inflammation factors were ameliorated when treated with sEH inhibitors (Stevenson et al., 2019). In another HF model induced by LPS, the cardiac function was decreased, as evaluated by hemodynamic analysis and echocardiography, while sEH deficiency attenuated LPS-induced cardiac dysfunction. It was also revealed that sEH deficiency lowered levels of TNFα and MCP-1 cytokines induced by LPS (Samokhvalov et al., 2018), which suggested that these protective effects of EETs against HF were beneficial for inhibition of inflammatory responses.

### EETs and Angiogenesis in Cardiac Remodeling

Capillary density is a crucial factor controlling the development of cardiac remodeling. Vascular endothelial growth factor (VEGF) is an important angiogenic factor involved in the maintenance of myocardial capillary density. This process involves proliferation, invasion, migration of endothelial cells, and tube formation, which is termed angiogenesis. Overexpression of CYP2J2 increased the expression of VEGF and promoted angiogenesis in the heart after ischemia (Zhao et al., 2018). The effects of EETs on angiogenesis were confirmed by Xu et al. with sEH inhibitors. A study carried out by Xu et al. revealed that sEH inhibition promoted a dose-dependent migration and tube formation of endothelial cell from MI patients, which could be abolished by a PPAR-γ antagonist, GW9662.

The effects of EETs on angiogenesis were reviewed by Fleming (2007) and updated by Imig (2016). The underlying cell signaling was explored by *in vitro* studies. In cultured endothelial cells, both 11,12-EET and 14,15-EET treatment increased cell proliferation and the formation of a tube-like structure by activating the AKT and MAPK pathways, which were downstream of VEGF-stimulated angiogenesis (Yang et al., 2009). Despite VEGF, fibroblast growth factor-2 participated in the angiogenesis promoted by EETs (Zhang et al., 2006).

Cardiac remodeling is a complex process in the challenge of stimulus, including a lot of pathological changes. Among them, inflammation, apoptosis, cardiac hypertrophy, fibrosis, and angiogenesis are the major changes. These changes are independent and closely related with each other. Besides, there are some other pathology changes such as oxidative stress and ER stress, which also play important roles in the pathogenesis and progress of cardiac remodeling. Research work carried out in our laboratory showed that elevation of EETs by overexpression or treatment with 11,12-EET or 14,5-EET showed protective effects against oxidative stress and ER stress (Wang et al., 2014; He et al., 2015; Zhou et al., 2018).

In summary, elevation of endogenous EETs by overexpression of CYP2J2 and sEH inhibition, as well as EET analogs, showed protective effects against cardiac remodeling, which was mediated mainly by prohibiting inflammation, apoptosis, fibrosis, and cardiac hypertrophy and promoting angiogenesis.

## Drug Development-Related EETs Against Cardiac Remodeling

Given the pathophysiological role of EETs in cardiac remodeling, the increase in EET availability emerges as a new opportunity to prevent and treat HF. Elevation of EETs has been viewed as a potential strategy for drug development. However, the half-life of EETs is very short, and its solubility is poor, which limits its clinical use.

Over the past decades, sEH inhibitors have been shown to increase the levels of endogenous EETs and showed a potent effects against cardiac remodeling (Imig, 2019). The key point to developing effective inhibitors is to optimize the absorption, distribution, metabolism, and excretion *in vivo*, as well as ease of formulation. In the early stage, AUDA was synthesized and showed a protective role in developing HF (Merabet et al., 2012). However, the drug is difficult to dissolve in water and even in several organic solvents with poor metabolic stability. Subsequently, AEPU was synthesized instead of AUDA, which showed a higher water solubility and is able to pass through the cell membranes freely. Recently, scientists found two novel compounds, TPAU and *t*-AUCB. These drugs participated in the attenuation of cardiac remodeling via oral administration (Cervenka et al., 2015a). Among all these sEH inhibitors, TPAU and t-AUCB showed an advantage in water solubility and metabolic stability. Up to now, all the studies were carried out in rodents, and the translation of sEH inhibitors needs further studies.

As described above, it has been well learned that sEH played an important role in the metabolism of AA (one of omega-6 polyunsaturated fatty acid, PUFA) into EETs. Interestingly, inhibition of sEH also increased the metabolites of omega-3 PUFA [including epoxyeicosatetraenoic (EEQs) and epoxydocosapentaenoic acids (EDPs) and linoleic acid (epoxyoctadecanoic acids (EpOMEs)]. Cristina López-Vicario et al. proved that sEH inhibition by t-TUCB increased the level of EDP and EEQ, as well as the level of EETs (Lopez-Vicario et al., 2015). In 1979, the protective effects of omega-3 PUFA were firstly reported in Greenland Inuits, who had higher fish oil intake (Dyerberg and Bang, 1979). The metabolites of omega-3 PUFA, EDP and EEQ were reported to play a similar role in the health (Jamieson et al., 2017a). In addition, EpOMEs were found as the metabolites of linoleic acid by CYP enzymes, which could be further metabolized to dihydroxyoctadecenoic acid (DiHOME) by sEH (Newman et al., 2005). Recent studies showed that DiHOMEs might be harmful to cardiovascular system (Hildreth et al., 2020). Thus, beneficial effects of sEH inhibitors might be induced not only by EETs, but also the elevated EDPs and EEQs, as well as the reduction of DiHOMEs.

Besides, EETs analogs were designed and synthesized. Up to now, a series of approximately 50 EET analogs were developed (Campbell et al., 2017). Among them, EET-A and EET-B are most studied in cardiovascular system. Cardiac beneficial effects for EET analogs have been revealed in both pressure overload- and volumed overload-induced cardiac remodeling (Hye Khan et al., 2014; Neckar et al., 2019; Vackova et al., 2019a). In addition, EET agonist, NUDSA, which is another kind of EET analog, ameliorated cardiac failure induced by ischemia (Cao et al., 2015).

EETs is the metabolite of AA via CYP enzymes, especially CYP2J2. The protective effects of CYP2J2 overexpression on cardiac remodeling are validated previously (Wang et al., 2014; He et al., 2015; Yang et al., 2015). Interestingly, besides of the direct beneficial biological effects of increased EETs, recent study reported that CYP2J2 also played an important role in transcriptional programs in adult human cardiomyocytes. CYP2J2 silencing resulted in the expression change of the genes involved in ion channel signaling, development, extracellular matrix, as well as metabolism (Evangelista et al., 2020). There are maybe two methods to regulate CYP 2J2 derived EETs. One is overexpression of CYP 2J2 in the tissue directly by gene therapy utilizing several vectors, such as recombinant adeno associated virus system. This system is approached by U.S. Food and Drug Administration to treat several diseases (Gruntman and Flotte, 2018). On the other hand, with the development of computer technique, the molecular secret of CYP 2J2 could be deeply discovered and potential drugs enhancing their activities might be a potential strategy for therapeutic design (Das et al., 2020).

## Conclusion and Further Perspective

**FIGURE 2:**
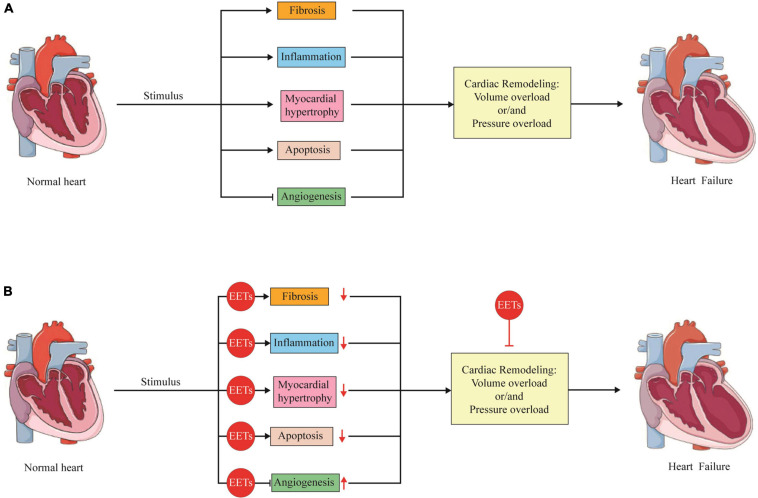
The role of EETs in the pathological changes of cardiac remodeling. **(A)** Under various stimulus, the heart will go through cardiac remodeling, which is an adaptive process to volume overload or/and pressure overload. During the process, several pathological changes including fibrosis, inflammation, myocardial hypertrophy, apoptosis as well as angiogenesis are involved, which finally caused heart failure. **(B)** EETs are able to attenuate cardiac remodeling with ameliorated myocardial hypertrophy, reduction in apoptosis and fibrosis, decreased inflammation as well as promotion in angiogenesis.

In summary, cardiac remodeling is the main pathogenesis of HF, which occurs in all kinds of CVD. Generally, there were two different models of cardiac remodeling depending on the overload. Pressure overload-induced cardiac remodeling is characterized with concentric hypertrophy, while volume overload mainly induces dicentric hypertrophy. In both cardiac remodeling, myocardial hypertrophy, apoptosis, fibrosis, inflammation as well as angiogenesis are involved. Once initiated, cardiac remodeling is hardly to be reversed and finally caused HF. In the past 20 years, EETs has attracted numerous attentions because of its protection effects against cardiac remodeling. A plenty of studies were carried out, and most results revealed that increase the level of EETs by different methods attenuated cardiac remodeling with ameliorated myocardial hypertrophy, reduction in apoptosis and fibrosis, decreased inflammation as well as promotion in angiogenesis (Figure 2).

Because of its instability, it is impossible for EETs to develop as exogenous drugs. Encouragingly, a lot of efforts have been made to explore the effective strategy to increase the level of endogenous EETs, including gene therapy, sEH inhibitor and EET analogs. Among them, sEH might be the most promising treatment. sEH inhibitor has been tested in a phase 2a clinical setting for its effectiveness in reducing blood pressure. Besides, the gene therapy has been largely developed recently, which could be prospective in upregulating the expression of CYP2J2 or deletion of EPHX2 in the future. Although, it is still a long way to translation into clinical use and further studies are needed to address these issues.

## Author Contributions

JL conceived and wrote the manuscript. CC supervised and wrote the manuscript. Both authors contributed to the article and approved the submitted version.

## Conflict of Interest

The authors declare that the research was conducted in the absence of any commercial or financial relationships that could be construed as a potential conflict of interest.

## Abbreviations

AA: arachidonic acids
ACF: aortocaval fistula
Ang II: angiotensin II
AMPK: adenosine 5’-monophosphate (AMP)-activated protein kinase
ANP: atrial natriuretic peptide
APOE: apolipoprotein E
AR: aortic regurgitation
ATF-6: activating transcription factor
BAX: BCL2-associated X
BCL2: B-cell lymphoma-2
BNP: brain natriuretic peptide
CAD: coronary artery disease
CHF: chronic heart failure
CHOP: C/EBP-homologous protein
COX: cyclooxygenase
CVD: cardiovascular disease
CYP: cytochrome P450
DHET: dihydroxyeicosatrienoic acid
DiHOME: dihydroxyoctadecenoic acid
EDHF: endothelium-derived hyperpolarizing factor
EDPs: epoxydocosapentaenoic acid
EEQs: epoxyeicosatetraenoic acid
EETs: epoxyeicosatrienoic acids
EpOMEs: epoxyoctadecanoic acids
ER: endoplasmic reticulum
ET-1: endothelin 1
GRP-78: glucose-regulated protein 78
HDL: high density lipoprotein
HETE: hydroxy eicosatetraenoic acid
HF: heart failure
HFD: high-fat diet
HO-1: heme oxygenase-1
ICAM-1: intercellular cell adhesion molecule 1
IGF: insulin-like growth factor
IL-1 α: interleukin 1 alpha
ISO: isoprenaline
JAK: Janus kinase
LAD: left anterior descending
LDL: low-density lipoprotein
LOX: lipoxygenase
LPS: lipopolysaccharide
LTX: leukotoxin
MAPK: mitogen-activated protein kinase
MI: myocardial infarction
MMP9: matrix metalloproteinase 9
MR: mitral regurgitation
NADPH: nicotinamide adenine dinucleotide phosphate
NFAT: nuclear factor of activated T cells
NF- κ B: nuclear factor kappa B
NO: nitric oxide
NUDSA: e(*S*)-2-(11-(nonyloxy)undec-8(*Z*)-enamido)succinic acid
PI3K: phosphatidylinositol 3 kinase
PLA: phospholipase
PPAR- γ: peroxisome proliferator-activated receptor gamma
rAAV: recombinant adeno-associated virus
RAS: renin–angiotensin system
ROS: reactive oxygen species
sEH: soluble epoxide hydrolase
STAT: signal transducers and activators of transcription
TAC: transverse aortic constriction
TGF- β: transforming growth factor beta
TNF- α: tumor necrosis factor alpha
VEGF: vascular endothelial growth factor.
